# Application of Aptamer-Based Assays to the Diagnosis of Arboviruses Important for Public Health in Brazil

**DOI:** 10.3390/ijms22010159

**Published:** 2020-12-26

**Authors:** Ana Paula Corrêa Argondizzo, Dilson Silva, Sotiris Missailidis

**Affiliations:** 1 Instituto de Tecnologia em Imunobiológicos (Bio-Manguinhos), Fundação Oswaldo Cruz, Av. Brasil, 4365-Rio de Janeiro/RJ CEP 21040-900, Brazil; acorrea@bio.fiocruz.br (A.P.C.A.); dilson.silva@bio.fiocruz.br (D.S.); 2 Programa de Pós-Graduação em Ciências Médicas da Universidade do Estado do Rio de Janeiro, Rua São Francisco Xavier, 255-Rio de Janeiro/RJ-CEP 22783-127, Brazil

**Keywords:** aptamer, arbovirus, diagnostic, therapeutic, dengue, Zika, biosensors, nanotechnology

## Abstract

Arbovirus infections represent a global public health problem, and recent epidemics of yellow fever, dengue, and Zika have shown their critical importance in Brazil and worldwide. Whilst a major effort for vaccination programs has been in the spotlight, a number of aptamer approaches have been proposed in a complementary manner, offering the possibility of differential diagnosis between these arboviruses, which often present similar clinical symptoms, as well as the potential for a treatment option when no other alternative is available. In this review, we aim to provide a background on arbovirus, with a basic description of the main viral classes and the disease they cause, using the Brazilian context to build a comprehensive understanding of their role on a global scale. Subsequently, we offer an exhaustive revision of the diagnostic and therapeutic approaches offered by aptamers against arboviruses. We demonstrate how these promising reagents could help in the clinical diagnosis of this group of viruses, their use in a range of diagnostic formats, from biosensors to serological testing, and we give a short review on the potential approaches for novel aptamer-based antiviral treatment options against different arboviral diseases.

## 1. Introduction

This review aims to cover the applications of aptamers as one among other strategies to face the great challenge offered by arboviruses in terms of public health in Brazil and worldwide. The use of such, not so new, tools against targets of a wide variety of molecular and biological natures has been increasing, and aptamer technology is establishing itself as one of the most significant branches of biological nanoparticle science. We start by defining arboviruses and their significance to human health, focusing our discussion on the current problems related to differential diagnosis. We subsequently provide an overview of their epidemiological panel, with emphasis on the context they have in Brazil, some cellular and molecular aspects related to viral metabolism, followed by a brief consideration of aptamer theory and technology, before finally discussing their various applications in arboviruses.

## 2. Arboviruses of Significance to Public Health

Arthropod-borne viruses, named arboviruses, are an important global public health threat. They cause disease in humans after circulating among wild animals and subsequently being transmitted to domestic animals that act as hosts [1]. Dengue (DENV) and chikungunya (CHIKV) viruses are two exemplar cases that have turned from an enzootic to an epidemic scale in Brazil and other important tropical urban centers.

Arbovirus is a term that does not denote a taxonomy classification, but includes a wide variety of RNA virus taxes, such as the alphaviruses from the family *Togaviridae*; the flaviviruses from the family *Flaviviridae*; the bunyaviruses, nairoviruses, and phleboviruses, all from the family *Bunyaviridae*; the orbiviruses, family *Reoviridae*; the vesiculoviruses, family *Rhabdoviridae*; the thogotoviruses from the family *Orthomyxoviridae* [1].

Arboviruses may be transmitted in two ways, although, by definition, they must replicate in an arthropod vector prior to transmission. They are mainly transmitted by biological transmission; the other mode of transmission, mechanical transmission, may occur through contaminated biological fluids. Details could be widely discussed here about the important aspects of viral transmission in order to better understand the peculiarities of epidemic spread. However, as this is not the focus of the review, we would only like to present some important aspects of the theme. Biological transmission can either be vertical (when the virus passes from an infected female vector to both male and female offspring) or horizontal (through the venereal way). It could also occur vertically from an infected male directly to a female vector. However, the main route is an oral transmission from a vector to a vertebrate host by injection of infectious saliva during blood feeding [1,2].

Arboviruses are RNA viruses, with the exception of the African swine fever virus (*Asfaviridae; Asfavirus*), which is the only known DNA arbovirus. This variety of RNA genomes and the different replication strategies adopted by these types of viruses demonstrate that the transmission through arthropods has been evolutionarily selected a number of times [3,4,5]. The genetic choice of RNA over DNA is an indication that the higher ability to mutate and the plasticity of RNA permit alternation in replication procedures in different vertebrate and invertebrate hosts [6].

Rapid climate change and manmade effects such as deforestation and migration of populations, lack of proper sanitary conditions, or expansion of urban areas without proper control and planning can result in increased transmission of arboviruses. There have been reports of West Nile virus (WNV) transmission in blood transfusions as well as organ transplantation procedures, but this ability to trigger iatrogenic infections is a characteristic of any viremia-producing arbovirus [2]. This is an additional factor that makes the medical importance of arboviruses evident and exemplifies the importance of early and differential diagnosis.

Brazil is a tropical country with a great terrestrial extension (8,512,000 km^2^) covered by forests, especially in the Amazon region [2,7]. The predominant climate of the country creates a favorable environment for the existence of arbovirus transmission vectors. Furthermore, the more than 200 million inhabitants of Brazil principally occupy middle and large-size urban centers in the south and north parts of the country, areas that are infected by the *Aedes aegypti* mosquito, the principal vector of a number of arboviruses. Under these favorable conditions, dengue has become an endemic disease and, on a number of occasions, caused large urban outbreaks. Similarly, yellow fever has caused a number of recent outbreaks, and its potential urbanization is a constant threat, potentialized by the movement of populations from sylvatic transmission areas to large urban centers [7]. Clinical manifestations of arboviruses in humans can range from undifferentiated, moderate, or severe febrile disease, rashes and arthralgia, and neurological and hemorrhagic syndromes. The febrile illness is usually accompanied by fever, headache, retro-orbital pain, and myalgia, whereas neurological syndromes can include meningitis, myelitis, encephalitis, paralysis, paresis, seizures, or problems associated with coordination or behavioral changes. Arthralgia manifests as a rash or maculopapular rash, polyarthralgia, and polyarthritis, whereas hemorrhagic syndrome is evidenced by petechiae, hemorrhage, and shock, combined with an intense reduction of platelets [2].

Among the most studied arboviruses are the viruses of the families Flaviviridae (DENV, Zika virus—ZIKV, yellow fever virus—YFV, Saint Louis encephalitis virus—SLEV, WNV, Rocio, Cacipacore, Ilheus, Bussuquara, and Iguape viruses), Togaviridae (Mayaro virus—MAYV, CHIKV, and eastern equine encephalitis virus), and Peribunyaviridae (Oropouche virus—OROV).

In the Flaviviridae family, the *Flavivirus* genus includes about 39 species that are classified as arboviruses, with some being the cause of encephalitis, and others provoking hemorrhagic fevers in humans and animals. The flaviviruses have a viral particle measuring 40 to 60 nm in diameter, with an icosahedral symmetry protein capsid enveloped by a lipid envelope where the membrane proteins and spikes of glycoprotein are inserted. The genome is a single RNA strand with the positive polarity of about 11 kb, and it encodes three structural proteins and five nonstructural (NS) proteins as follows: 5’-C-preM-E-NS1-ns2a-ns2b-NS3-ns4a-ns4b-NS5-3’, which play a role in regulating and expressing the virus, such as replication, virulence, and pathogenicity [2].

DENV virus is the most important flavivirus causing human disease in Brazil, and it is represented by four serotypes (DENV-1 to DENV-4). Viruses are kept in wild cycles involving small primates and mosquitoes of the genus *Aedes*. Its transmission is mainly through the mosquito *A. aegypti*, but *A. albopictus* and *A. polynesiensis* are secondary vectors. The main transmission cycle of DENV involves only humans and mosquitoes in large, tropical, urban centers. The disease presents with symptomatology ranging from fever to hemorrhagic fever or dengue shock syndrome. Other symptoms can be related to pain in the form of myalgia, headache or retro-orbital pain, or bleeding, which, apart from the hemorrhagic fever, can include petechia, epistaxis, and bleeding of the gingiva [2,7].

Another arbovirus that is widely disseminated in the Americas is SLEV, which is found in a number of countries from Canada to Argentina. SLEV is a neurotropic virus that, at least in the Amazon region of Brazil, is found to use *Culex declarator* and *C. coronator* as vectors. Its reservoirs include various animal species such as birds, monkeys, sloths, armadillos, and marsupials [7]. The disease associated with SLEV can range from fever and headache in mild cases to meningitis and encephalitis in severe cases [2].

Rocio virus is also neurotropic in nature and can cause anything from asymptomatic infection to acute encephalitis, with an incubation period of 7 to 14 days. The symptoms are acute fever, headache, anorexia, nausea, vomiting, myalgia, and malaise. Signs of encephalitis are late and include mental confusion, reflex disturbances, motor disability, meningeal irritation, and cerebellar syndrome. Patients may also present seizures, abdominal distension, urinary retention, visual, olfactory, and auditory disorders, lack of motor coordination, impaired balance and swallowing, and memory disorders [8]. The Rocio virus was isolated for the first time during an outbreak of encephalitis in the state of São Paulo, in the Atlantic forest zone of the Ribeira Valley, in 1975. About 1000 cases of encephalitis and a 10% mortality rate were registered during the seven years of this outbreak. Studies on the Rocio virus have identified migratory and other wild bird species as reservoirs, whereas the vectors of this virus are considered the *Aedes* and *Psorophora* mosquitoes [2,7].

WNV was first isolated in the West Nile province of Uganda in 1937. The WNV transmission cycle involves mosquitoes and birds, and humans are an accidental host. About 80% of infected people are asymptomatic. Of those with symptoms, about 20% develop the characteristic febrile illness, with an incubation period of 2 to 14 days, causing fever, headache, fatigue, rash, palpable lymph nodes, and ocular pain. The most serious manifestations are meningitis and encephalitis [9].

Another flavivirus is the Japanese encephalitis virus (JEV) that is considered to have its origin in Indonesia and Malaysia from where it has spread widely in Asia and as far as Australia to become the major cause of arbovirus-related encephalitis. Children and adolescents are mostly affected by this virus and constitute 75% of the registered cases. JEV presents a high fatality rate, which can be up to 30% in severe cases, but it is also the cause of permanent neurologic and/or psychiatric damage to some 30–50% of the victims. Whilst humans and horses can be infected by the virus, they are not able to infect mosquitoes due to the low viremia developed during the infection [10].

Yellow fever is an infectious, noncontagious disease caused by YFV in two basic cycles: an urban human–mosquito cycle in which *A. aegypti* is the main transmission vector, and the more complex wild cycle, where a broad range of mosquitoes can cause virus transmission. These include, in Africa, mosquitoes *Aedes* (*A. africanus, A. furcifer,* and *A. simpsoni*), and in America, mosquitoes *Haemagogus (H. janthinomys, H. albomaculatus, H. leucocelaenus*) and *Sabethe schloropterus* [11]. Whilst YFV can be the cause of severe disease in 10% of the cases, and in those, reach a fatality rate of 50%, it remains mild or asymptomatic in the majority (nearly 90%) of the cases. Africa is most affected by yellow fever, and urban epidemics still appear in these regions [11]. The clinical manifestations of yellow fever vary from slight (fever and headache), moderate (previous symptoms, including myalgia, arthralgia, nausea, vomiting, and asthenia), severe (previous symptoms, including jaundice, hematemesis, or oliguria) and malignant (all classic symptoms observed).

ZIKV gets its name from the Zika forest in Uganda, where the virus was first identified in a rhesus monkey in 1947. This was followed a few years later by the first reported infection in humans in the Macnamara region of Nigeria, in 1954, and the isolation of the virus in *A. aegypti* mosquitoes [12,13]. The same *Aedes* species was the origin of the first isolation of ZIKV in Asia, in Malaysia (1969) [13,14]. As with other arboviruses, the majority of ZIKV cases remain asymptomatic or express mild symptoms. However, ZIKV has been responsible for Guillain–Barre syndrome and meningoencephalitis in adults, though the biggest threat it represents is the causing of microcephaly and other neurological damage in infants due to the passing of the virus from pregnant women to the fetus [15]. ZIKV demonstrates both an enzootic, sylvatic cycle, with viral circulation between the *Aedes* spp arboreal mosquitoes and nonhuman primates, and an urban cycle that is distinct both ecologically and evolutionarily between humans and peridomestic or domestic mosquitoes of the *Aedes* spp [16]. It takes 3–11 days after infection for the initial symptoms to appear, and they are usually manifested in the form of a mild headache, followed by a maculopapular rash, malaise, arthralgia, conjunctivitis, and other symptoms that can vary between patients. Thus, diarrhea or constipation, anorexia and abdominal pain, or dizziness have been reported as well as rash, although these are not characterized as common features. Myalgia, vomiting, or retro-orbital pain have also been reported, but these are considered less frequent disease manifestations [17].

An overview of emerging arboviruses also brings us other, less distributed and more localized diseases: (a) Bussuquara virus, first isolated in the state of Pará in the Amazon region of Brazil in 1956, from sentinel and wild animals such as the *Proechimys* spp. rodents or *Culex* mosquitoes. Serological investigations suggest that it is an infection of rare occurrence, with a single case of reported human febrile infection. (b) Cacipacore virus, first isolated in 1977 from a bird, also in the Amazon region (state of Pará, Brazil), but, to date, not associated with any disease in humans and no vector has yet been identified as responsible for its transmission. (c) Iguape virus, isolated in 1979 from a sentinel mouse in the coastal area of the rainforest of southeastern Brazil. Human diseases caused by Iguape are still unknown. (d) Ilhéus virus, isolated for the first time in 1944 from a pool of *Aedes* and *Psorophora* mosquitoes; it causes infection in humans and is characterized by severe headaches, chills, myalgia, and weakness, with or without mild encephalitis syndrome [2,7].

In the Peribunyaviridae family, genus *Orthobunyavirus*, we have OROV. This virus is transmitted between sloths, marsupials, primates, and birds by the mosquito *A. serratus* and *Culex quinquefasciatus*. Remarkably, this virus has adapted to an urban cycle involving humans, with *Culicoides* mosquitoes from Pará (Brazil) as the main vector. OROV has already caused large outbreaks of acute febrile disease in the Amazon and central region of Brazil, with an estimated 500,000 cases of infection by this virus in Brazil in the last 48 years [7,18,19]. The medical importance of OROV has recently become evident in the regions of the Amazon basin of northern Brazil and Peru. In the period from 1980 to 2004, in Brazil, the virus spread to the states of Amazonas, Amapá, Acre, Rondônia, Tocantins, and Maranhão. In 2000, one strain was isolated from a new host, the monkey *Callithrix* sp. in the state of Minas Gerais [18]. Although OROV is the second most frequent arbovirus to cause febrile illnesses in Brazil, little is known about the pathogenesis of infection. Experimental infections, established in golden hamsters, have shown that about 50% of the animals studied develop disease characterized by lethargy, chills, tremors, paralysis, and about one-third die with high viral titers in the blood, liver, and brain [7].

Rift Valley fever virus (RVFV), *Phlebovirus* genus of the Peribunyaviridae family, is endemic to the sub-Saharan region of Africa and has been reported to have spread to Egypt and become endemic to the whole of the Arabian Peninsula. This virus presents a number of symptoms that, in the cases of severe disease, can include hepatitis, encephalitis, and hemorrhagic fever. RVFV has further economic significance as it is responsible for abortion storms in livestock [20,21]. The nucleocapsid protein is one of seven proteins encoded by the viral RNA genome. This protein is of particular importance due to its participation in the protection of the viral genome from degradation, the prevention of double-stranded RNA formation during replication and transcription, the removal of higher-order RNA structures that could have a negative effect on the rapid and efficient mRNA translation, and through its direct participation on the initiation phase of replication, functioning as an RNA chaperone [22,23]. As has been reported for ZIKV, RVFV can also be transmitted through contaminated biological fluids such as blood, milk, and tissue, as well as the bite of RVFV-infected mosquitos, particularly of the Culicidae family [24,25]. In fact, some 73 species in the 8 genera of this family have been shown to be competent transmission vectors of RVFV. Heavy rain periods are often followed by enzootics/epidemics of RVFV in endemic areas as they cause an increase in the mosquito vectors due to the favorable breeding conditions created. Additionally, whilst horizontal transmission is favored in such interepidemic periods between mosquitos and farm or wildlife reservoirs, vertical transovarian transmission has also been described [25,26].

In the Togaviridae family, *Alphavirus* genus, representative arboviruses are the MAYV, CHIKV, and eastern equine encephalitis viruses. MAYV was first isolated in 1954 from febrile workers in Trinidad. Subsequently, several individuals with fever and headache from the area of the Guamá River, state of Pará, Brazil, were also identified as infected by the virus. Several fever and arthralgia outbreaks in the Amazon region and the central plateau of Brazil and in various other Latin American countries have been attributed to MAYV. The main vectors are forest-inhabiting mosquitoes of the genus *Haemagogus*, and the vertebrate hosts are mammals. Symptoms associated with MAYV include fever, headache, myalgia, arthralgia, and, at times, arthritis. As has been described above with a number of other arboviruses such as dengue and RVFV, the rainy season increases MAYV occurrence, although MAYV illness persists throughout the year. The estimated transmission of MAYV in the city of Manaus alone is more than 2 million people, making MAYV a relevant public health problem, especially as control of the vector is nonviable and there is no vaccine or treatment available [2,27].

Another zoonotic arbovirus of the Togaviridae family is CHIKV, which was initially isolated in Africa in 1952 from a contaminated primate. *A. aegypti* and *A. albopictus* are the described vectors of CHIKV in the urban cycles, where symptoms appear 2–4 days postinfection and viremia in a further 5 days after the first symptoms appear. Typical symptoms of CHIKV include fever, as with other arboviruses, with common manifestations of arthralgia, backache, and headache. A maculopapular rash can also manifest as a symptom some days after the initiation of the disease and can last up to 10 days, as well as neuroretinitis or sand uveitis. More rare neurological diseases can also be present in the form of febrile seizures, meningitis, or encephalitis [28].

It thus becomes clear that arboviruses are of great relevance to public health due to a number of factors, from the diversity of the infectious agents involved and the plurality of clinical manifestations to the lack of efficient differential laboratory diagnosis, lack of immunoprophylactic measures for most of the current infections, and the difficulty in implementing and maintaining educational and sanitary measures. Consequently, new approaches for rapid laboratory diagnosis and effective control and prevention of these infections are urgently required. Appropriate diagnosis is the key factor for the treatment of viral diseases when these infections are difficult to distinguish clinically. Especially at the onset, when the infection appears, patients present nonspecific signs and symptoms, often common in many of these diseases. Finally, due to their antigenic similarity, it is difficult to distinguish among some of these infections, even with serological tests, due to several cross-reactions among them.

## 3. Current Problems with Differential Arbovirus Diagnosis

Arboviral diagnosis is essentially a great challenge, whether in clinical examination or in laboratory assessment, due to the particular characteristics of each set of symptoms, which could be present or not, the similarities between one or more symptoms, and the phylogenetic cross-reaction in serological tests that often occur, especially in endemic regions [29].

Specificity is probably a major question related to most arbovirus diagnostic tests and it plays an important role in the differential identification of these ever-growing diseases. Many methods and strategies have been proposed to better discriminate these viruses, ranging from tests using traditional equipment for molecular diagnostic assays, supported by the high reliability of RT–PCR, nested RT–PCR and real-time RT–PCR machines to the use of point of care (PoC) immunochromatographic tests and novel biosensors.

As discussed above, the majority of the arboviruses, at least at the early stages of the infection and during the light and medium stages of symptoms, present a clinical profile that is very similar, namely, fever, rush, headaches, and fatigue, that in an endemic area like Brazil could indicate any number of viruses. In addition, whilst molecular diagnostics are accurate, they are expensive, require specialized equipment and trained personnel, and are available only in large medical centers and central laboratory facilities. In the case of Brazil, as well as in many other tropical countries, this would also be affected by the logistics of shipping samples, maintaining a cold chain, and delays in processing and analyses. Finally, molecular diagnosis is only possible during the acute phase of the disease, which is during the first few days postinfection. On the other hand, serological tests are often incapable of specifically identifying antibodies from viruses of the same genus, particularly in the case of immunoglobulin G (IgG). Thus, whilst serological testing for immunoglobulin M (IgM) is reasonably accurate, IgG between dengue and Zika or Chikungunya and Mayaro is practically indistinguishable due to the cross-reactivity of such antibodies, giving false results and masking epidemiological data.

## 4. Aptamer-Based Diagnostic Technology

**Figure 1:**
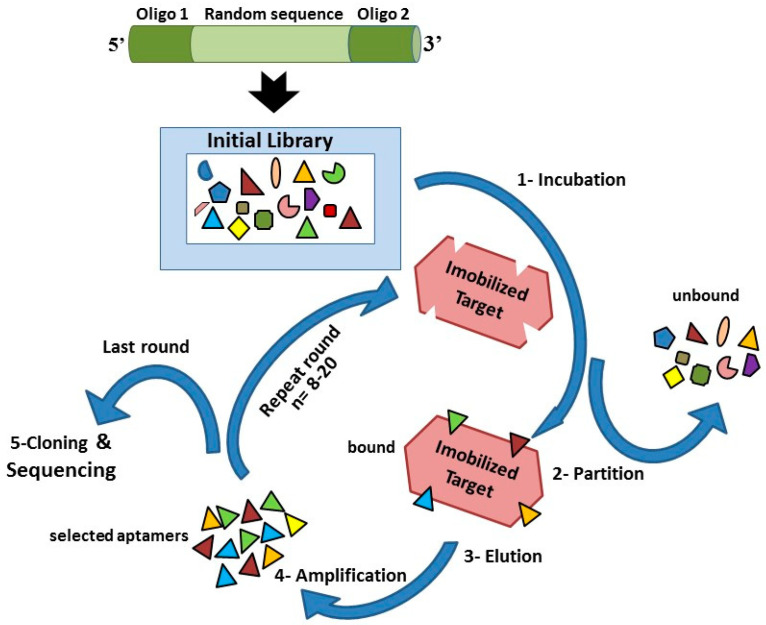
The SELEX (systematic evolution of ligands by exponential enrichment) scheme. (1) Incubation of the initial library with specific target, (2) partition of bound from unbound sequences, (3) elution of bound sequences, (4) amplification of eluted sequences, and (5) cloning and sequencing.

In the last 25 years, aptamers and SELEX (systematic evolution of ligands by exponential enrichment) technology have received great attention. Aptamers are single-stranded nucleic acid molecules consisting of DNA or RNA of about 20 to 80 bases, with molecular weights of 6 to 30 kDa, that adopt various three-dimensional conformations that bind to organic or nonorganic molecules—from single atoms to a wide range of proteins, as well as more complex targets such as whole cells, viruses, or bacteria. Aptamers are characterized by high specificity to target molecules and high binding affinity, inherent to the way they are selected. Thus, the dissociation constant of the aptamer/target molecule complex is frequently in the nanomolar or picomolar range [30]. The SELEX technique was independently invented in 1990 by two teams, Ellington and Szostak and Tuerk and Gold, and has remained dominant since the discovery of aptamers, although it has been subjected to a number of variations to its original protocols [31,32]. The SELEX methodology remains the gold standard for the development of specific aptamers, and the process includes several rounds of amplification and enrichment that allow the evolution of aptamers with high affinity, specific to a given target, from a set of random oligonucleotides. Usually, negative selections (counterselections) are added to the methodology and allow the elimination of nonspecific sequences that also bind to similar or related targets [33,34]. Thus, specific aptamers can be generated after about 8 to 20 selection cycles, as shown in Figure 1.

Similar to the conformational recognition that mediates antibody–antigen recognition and the formation of complexes, aptamers bind to their cognate targets with high specificity and affinity through various types of interaction, such as Van der Waals forces, hydrogen bonds, electrostatic interactions, and bonds between the complementary bases. Thus, aptamers are also referred to as “chemical antibodies” and are functionally used as antagonists, agonists, or target ligands and are applied in a variety of bioanalytical and biomedical assays, as well as therapeutic applications [33,35].

Aptamers are excellent alternatives to monoclonal antibodies, which have high production costs and important limitations such as temperature instability or low secretion by the obtained clone. Therefore, it becomes apparent that the affinity and specificity of these molecules position them as an important technology, not only for diagnostic and therapeutic purposes but also for bioanalytical applications [33,36,37,38]. In addition, aptamers are synthetic molecules and have reduced size and nucleic acid characteristics, such as thermal stability, which may improve their applicability and suitability for industrialization. Moreover, chemical modifications may be easily implemented on a large scale, with minimum lot-to-lot variations. Aptamers also have a potentially superior clinical applicability to antibodies in several respects: (i) they are practically nonimmunogenic and nontoxic in vivo; (ii) because of their smaller size, they have superior tissue penetration, increasing their therapeutic index; (iii) they can be developed against a seemingly limitless range of targets. Thus, there are several studies showing that these molecules have already been developed against small inorganic ions, drugs, peptides, proteins, and complex cells or tissues, demonstrating the wide scope of aptamer applications [39].

In order to address the problem of nuclease stability, modifications are often employed in order to stabilize the aptamers, including the design of spiegelmers or the chemical modification of either the oligonucleotide backbone or the 2′-position of the pyrimidine moiety. Mairal et al. [33] cited several works in their review that employed methods like the use of modified deoxynucleoside or ribonucleoside triphosphates during the selection process, the use of α-thio-substituted deoxynucleoside triphosphates using a phosphorothioate DNA library, or modification of the 2-position of pyrimidine nucleotides with amino/fluoro groups as a means of stabilizing aptamers. Spiegelmers, on the other hand, are an alternative route to producing nuclease-resistant aptamers because they are synthetic mirror-image RNA or DNA oligonucleotide ligands that are based on the unnatural enantiomeric form of the sugars ribose and deoxyribose. They can bind specifically to a target but are not recognized by ribonucleases, thus presenting high biological stability and long life.

Appropriate diagnosis is important for defining medical care options in viral disease patients. However, viral infections, especially during the acute phase, often cause common, nonspecific symptoms that confuse the medical diagnosis. Pan et al. [34] presented a major revision on the use of antibacterial aptamers for the diagnosis of bacteria like *Mycobacterium tuberculosis* and *Staphylococcus aureus*, among others. Despite the fact that we will not describe their work here because the main subject of their study is not within the scope of our review, these authors also listed a series of potential antiviral aptamers for therapy. Similar to Pan et al. [34], other studies have mostly focused on a few well-known viruses, such as the human immunodeficiency virus type 1 (HIV-1), hepatitis B virus (HBV), hepatitis C virus (HCV), human papillomavirus (HPV), severe acute respiratory syndrome (SARS), and influenza. Thus, the development of aptamers as diagnostic or therapeutic agents against other viruses has been limited and focused around individual groups, as has been the case with aptamers against arboviruses [30,40]. In this review on the subject, we focus on the specific works for the diagnosis of arboviruses based on aptasensors.

## 5. Application of Aptamer-Based Assays to the Diagnosis of Arboviruses

Rapid population growth and the increased resistance to or lack of effective treatment of many viruses creates an obvious need for effective diagnosis, the ability to differentiate with high sensitivity and specificity between different viruses or other pathogens. One such rapidly growing area in viral diagnosis is based on biosensor technology, where aptamers can play an important role as recognition elements. Their low production cost compared to antibodies, their equal or better sensitivity and specificity, and their superior thermal stability, which eliminates the need for cold chain, are some characteristics that could make aptamers an important part of biosensor development. Aptamers can be a financially viable diagnostic solution during the acute phase of the disease, for the recognition of viral antigens soon after infection, as well as to monitor disease progression during treatment. SELEX offers a versatile and rapid PoC platform for aptamer selection, and the ease of chemical modification to further increase selective pressure could result in aptamers characterized by a precise detection capability of viremia levels that are well below those of currently available diagnostic assays [30,39].

Many strategies have been adopted in the use of aptamers, either in diagnostic or therapeutic products against arboviruses, but until now, the literature has focused mainly on ZIKV and DENV diagnostics, with some studies on the use of aptamers for diagnosis of tick-borne encephalitis virus (TBEV), RVFV, and CHIKV, among others.

With regards to the use of aptamers in the diagnosis of arboviruses, Bruno et al. [41] demonstrated the potential for DNA aptamer selection using commercially available sources of viral envelope proteins or synthetic peptide-based antigens representing epitopes of such proteins. These aptamers offer a potential diagnostic assay alternative and the possibility to be used in a rapid PoC arbovirus diagnosis test format. In this work [28], generated DNA aptamers were directed against the following targets: CHIKV E1a peptide, Crimean Congo hemorrhagic fever (CCHF) Altamura Gn 611, 11E7a, 11E7b, and 11E7c peptides, recombinant dengue type 1 to 4 envelope (E) antigens, TBEV CE/gE protein, and WNV E protein. The aptamers generated were tested in enzyme-linked aptamer sorbent assay (ELASA) affinity screening, and the top 10 aptamers for each virus were ranked based on absorbance intensity and subsequently used in further studies. The best of the selected aptamers in terms of affinity were used in lateral flow (LF)-based assays, or in fluorescent sandwich tests on the surface of magnetic beads. Using the CHIKV E protein as a target, the group demonstrated that two different aptamers could successfully work as a pair in an LF sandwich assay, one as a conjugate and one as a capture agent. For TBEV, it was demonstrated that one selected aptamer could be conjugated to a gold colloidal, whilst a different TBEV aptamer could be used in the capture dot, permitting the detection of the TBEV envelope protein. This proved the feasibility of LF-type assay development using aptamers for the detection of arboviral proteins. Similarly, the highest affinity and best-studied CCHF aptamers were used in the development of a fluorescent aptamer–MB sandwich assay as further proof of their commercial potential. Different combinations were tested to assess the best pair of aptamers for the application, and superior fluorescence detection was observed with the Gn6-25R aptamer as a capture agent and the Gn6-17F-TYE665 as a reporter. This work demonstrated that aptamers performed well both in LF and fluorescent sandwich assays and could, therefore, be used for the detection of arboviruses under such conditions.

Ellenbecker et al. [21] described the selection and characterization of RNA aptamers for the nucleocapsid (N) protein of RVFV. The recombinant N His-tag protein was expressed in *Escherichia coli* and purified in a Ni-charged resin from the cell lysate. Subsequently, a SELEX-type selection was performed to isolate families of RNA aptamers with binding properties characterized by high affinity and specificity for the target protein. The group identified GAUU and pyrimidine/guanine motifs present in both their selected aptamer sequences and in the coding region of the RVFV viral genome. A selected and subsequently truncated aptamer, labeled with fluorescein and studied in a fluorescence polarization system during a titration experiment with the target protein, demonstrated a high-affinity binding with a Kd of 2.6 µM, offering data that support the possibility of constructing a sensitive fluorescence-based sensor for the viral N protein, thus permitting viral testing or drug screening application development.

For the development of therapeutics against TBEV, Kondratov et al. [42] obtained aptamers to a fragment of surface protein E of this virus. The recombinant His-tag E protein was expressed in *E. coli* and purified in a Ni-charged resin. The purified recombinant protein was immobilized on immuno plates, where 15 rounds of a SELEX-based selection of aptamers were performed. The antiviral activity of the pool of aptamers was assessed using an isolate of the Siberian TBEV in pig embryo kidney cell (PK cell) culture. The concentration of infectious virus (PFU/mL—plaque-forming unit/mL) was determined by titration of the PFU in PK cell culture, and the neutralization index was calculated as the ratio between the titers of viral suspensions in the control and after the treatment with the pool of aptamers. As a result of the treatment with the pool of aptamers, the TBEV neutralization index *I* averaged log*I* = 1.16 ± 0.05, i.e., the amount of infectious virus in the suspension decreased approximately 17.5 times. This value is comparable with the results of the neutralization of the commercial human immunoglobulin against tick-borne encephalitis at a dilution of 1:80 (log*I* = 1.2–1.88). Since the analyzed pool apparently contained groups of aptamers with different inhibitory activity, it can be expected that this parameter will increase after additional rounds of selection. Aptamers specific for TBEV surface protein can also be used in diagnostic platforms. Today, enzyme immunoassay systems based on the immobilization of viral particles on antibodies are most commonly used for TBEV diagnosis. However, after obtaining a positive result, it is impossible to isolate the virus in physiological conditions for further research. On the other hand, if the virus is immobilized on aptamers, the virion–aptamer complex can be easily destroyed with deoxyribonuclease, and the probability of isolating intact viral particles is very high. In this case, the aptamers can be used to isolate live virus from natural and clinical specimens.

In relation to DENV diagnostics, Fletcher et al. [43] demonstrated a modular biosensor able to rapidly identify sequences associated with the genome of the dengue virus. As described by the authors, the biosensor consists of an oligonucleotide linker module, an aptamer/restriction endonuclease signal transducer, and a fluorescent signaling molecule. The conformation of the linker molecule is in the form of a simple stem/loop, which comprises a target–complementary moiety within the loop and a trigger moiety within the stem. When bound to the target nucleic acid, the trigger strand of the denatured stem can bind to the aptamer within the signal transducer. Disruption of the aptamer releases the restriction endonuclease *Eco*RI from aptamer-mediated inhibition. Active *Eco*RI is able to rapidly cleave multiple signaling molecules to generate a detectable signal. Upon analysis, a highly specific binding of the linker to its nucleic acid target was shown, and this quickly resulted in a distinct fluorescent signal. Furthermore, such a signal could be clearly distinguished from the control signal produced by nontarget sequences within 20 min. Moreover, when serotype-specific linkers were used, the resulted signal by different dengue serotypes was equal to that of the negative control non-dengue sequences. Thus, it was verified that serotype-specific aptamers were developed, and the test did not present any cross-reactivity between different dengue serotypes. When a linker capable of identifying all dengue serotypes was used, the test was found capable of distinguishing between dengue and non-dengue sequences. Thus, the biosensor developed was capable of specifically detecting and distinguishing between dengue serotypes with high specificity, as well as recognizing a pan-dengue sequence. Finally, apart from the efficiency of the described biosensor, the author pointed out its robustness and the potential low-cost of its reagents as a means of its viability in product development for the detection and control of infectious diseases.

Gandham et al. [44] described the selection of thioaptamers targeting dengue virus type-2 envelope protein domain III (EDIII) that may be used as an antiviral drug. In this work, the DENV-2 EDIII was cloned, expressed, and purified, and the recombinant protein used to generate a thioaptamer. To select the thioaptamers, a SELEX procedure was performed using magnetic beads coated with the recombinant protein. The four most abundant thioaptamers (DENTA 1–4) were synthesized with 5′-biotin ends and used in the characterization studies. Filter-binding assays demonstrated that the selected thioaptamers bound tightly to DENV-2 EDIII, with Kd ranging from 99 nM (DENTA-1) to 180 nM (DENTA-4). DENTA-1 binding to DENV-2 EDIII was measured using microscale thermophoresis (Kd = 154 ± 40 nM), where the binding was measured in solution. Using 2D HSQC NMR, the authors could decipher the protein residues perturbed by the interaction, thus identifying which ones were involved, directly or indirectly, with the aptamer binding. Consistent with previous antibody-related studies, where the residues F305–A312 were identified as the neutralizing epitope for the selected antibody 1A1D-2, the DENTA-1 aptamer also bound to this same epitope of EDIII and was shown to neutralize the virus. It is important to note that previous cryoelectron microscopy and crystal structure experiments have identified this DENV-2 EDIII epitope as an apparently not-surface-exposed region in the static structure of the virus, which, nevertheless, is a target for both neutralizing antibody and aptamer. Furthermore, using dentrimers or other molecular scaffold systems, it is possible to design molecules that can bind and lock the EDIII positions at the fivefold or threefold symmetry axes on the surface of the dengue virus. This consequently also increased the avidity of the selected aptamers, thus resulting in their increased neutralization capacity. This is a unique approach in terms of antiviral treatment and could lead to the design of potent viral inhibitors, especially in enveloped viruses where their surface symmetry can be adequately exploited to such means. Chen et al. [45] also demonstrated the selection and characterization of DNA aptamers targeting all four serotypes of dengue virus, with a potential application as antiviral drugs. The E protein of flavivirus is the better-chosen antigen in eliciting neutralizing antibodies as it induces a suitable immunologic response in the infected host and it is associated with the entry of DENVs into host cells and membrane effusion. From the three domains of the protein, EDI, EDII, and EDIII, the last one participates in the attachment of the virus membrane to the host cell membrane receptors, resulting in further internalization and membrane fusion. As suggested by studies with monoclonal antibodies, a good strategy to avoid the viral attachment is to block the ED3-targeting. In this work, the DNA fragments encoding part of DENV-2 EDIII were cloned and the recombinant His-tag protein was purified. EDIII mutants were generated by site-directed mutagenesis and were expressed and purified in the same manner as the wildtype EDIII protein. A total of 8 mutants of ED3 (I335A, R345A, Q316G, H317A, G318A, G318P, T319A, and I320A) was obtained. EDIII–ssDNA complex was eluted with imidazole, and ssDNA was amplified. The reverse primer was biotinylated, and PCR products were purified using a Streptavidin Ultralink Resin. A total of 15 rounds were performed. Interactions of aptamers and DENV-2 ED3 were evaluated by their Kd using the technique of fluorescence quenching, and the number of binding sites (n) was calculated by the Stern–Volmer equation. The parallel G-quadruplex structure was experimentally verified by circular dichroism. The aptamers–protein interaction was evaluated by ELASA and Western blot experiments. Two clones were selected, and in the fluorescence quenching analysis, the best fit was calculated for aptamer S15 by setting Kd = 200 nM and n = 1.14. Aptamer S22 had n = 1.09, and even with almost the same binding stoichiometry, its binding constant was significantly higher, with Kd = 500 nM. Aptamer S15 recognizes both DENV-2 virion and EDIII. The results of the plaque reduction neutralization test assay have shown that the infection of DENV-2 was completely neutralized by 100 μM S15. On the other hand, the 50% inhibitory concentration values for the neutralization activity against DENV-1 and DENV-3 serotypes have a similar range with that of DENV-2, but only DENV-2 can be completely neutralized by S15 at 100 μM. In addition, the S15 aptamer was found to form a parallel quadruplex structure that, together with the sequence on the 5′-end, was necessary for the binding activity to a highly conserved loop between A and B strands of EDIII. The authors provided the first aptamer with antiviral activity against the four serotypes of DENV and revealed the importance of residues on the loop between βA and βB strands of DENV EDIII for neutralizing the four dengue serotypes.

Balinsky et al. [46] have shown that the DENV capsid protein C interacts and colocalizes with the multifunctional host protein nucleolin. The aptamer AS1411 against the protein nucleolin, originally developed by Aptamera for the treatment of cancer, has been shown to interact and block its interaction with the DENV capsid protein C. Furthermore, in a treatment study, this aptamer has been shown to reduce viral loads postinfection. Finally, it has been shown that AS1411 is capable of altering the migration pattern of the virus, identifying nucleolin as a potential target in antiviral treatment design.

Alejo-Cancho et al. [47] described a new microfluidic DENV NS1 immunomagnetic agglutination assay (IMA) in a recent paper. It is based on the aggregation of magnetic nanoparticles detected by an electronic reader. This rapid and semiquantitative test was compared with another two: the SD Dengue NS1 Ag ELISA, for the qualitative detection of NS1 antigen in human serum, and the SD BIOLINE Dengue Duo kit, a rapid immunochromatographic test (ICT) that detects both DENV NS1 antigen and antibodies against DENV (IgM/IgG) in human serum, plasma, or whole blood. The IMA is a rapid and semiquantitative microfluidic DENV NS1 detection method based on magnetic nanoparticles coated with a mix of monoclonal antibodies capable of detecting NS1 from all four DENV serotypes. IMA technology has a limitation that has been described for NS1 detection assays, which refers to lower sensitivity for DENV-2 and lower detection of secondary dengue infections. In this work, a total of 135 serum samples from travelers returning from dengue-endemic countries were analyzed, including specific positive serums for dengue, Zika, Chikungunya, and malaria, as well as negative samples. The IMA test is a rapid test that uses little sample volume and provides results in 15 min. Thus, comparing this rapid test with the rapid test of the ICT type, it was found that IMA had a much better sensitivity (91.1%) when compared with the sensitivity demonstrated by ICT (68.1%). On the other hand, the specificity of IMA was slightly lower (98.4%) when compared to that of ICT, which was 100%. The best result was obtained with ELISA, which is the best test after the gold standard, which remains RT–PCR. ELISA demonstrated a sensitivity of 97.2% and specificity of 100%.

Basso et al. [48] described a new immunosensor using hybrid nanomaterials for the detection of the dengue virus. The immunosensor is composed of stoichiometric maghemite, γ-Fe_2_O_3_ (named surface active maghemite nanoparticles, SAMNs) modified with 3-mercaptopropionic acid–MPA (SAMN@MPA) and bound to gold nanoparticles (AuNPs) conjugated with aptamers, forming the SAMN@MPA@AuNPs@aptamer that is a colorimetric immunosensor. For a better understanding of how to obtain these particles, we recommend reading the original article. Specific aptamers for the four dengue serotypes were modified in the 5′-terminus with a -SH group for binding to AuNPs. A pool containing the four dengue serotypes (DENV-1, -2, -3, and -4) was added to the SAMNs@MPA@AuNPs@aptamer complex and a decrease in the intensity of the absorbance peak was observed. The aptamer–virus conjugation causes a reduction of the surface area of AuNPs, justifying the observed reduction of absorbance and the wavelength displacement of the localized surface plasmon resonance signal. In addition, it is possible to observe a color change at each step of the immunosensor production according to the surface modifications of the hybrid nanomaterial. Thus, SAMNs only present an earthy yellow coloration, and AuNPs a red coloration. The SAMNs@MPA@AuNPs@aptamer complex showed a dark purple coloration caused by the aggregation of the aptamers in the nanoparticles, and after the addition of the pool with the four dengue serotypes, the solution showed a change in its coloration to green, indicating the detection of the virus caused by the antigen–aptamer binding. Tests with ZIKV and YFV have shown no false-positive response. The method described represents an easy, fast, low-cost, and specific base to a test, which could be used in field measurements for the diagnosis of dengue.

The development of a Zika diagnostic assay was proposed using an aptamer-based ELASA for the highly specific and sensitive detection of Zika NS1 protein [15]. NS1 is a glycosylated protein and is significant for the effective emission, virulence, and replication of the virus. The protein may be in the form of a monomer, a dimer (a membrane-bound protein, mNS1), or a hexamer (a secreted protein, sNS1). The homodimer of NS1 plays a key role in viral replication, whilst membrane-bound NS1 is secreted and is responsible for provoking immune responses [17]. Aptamers were raised against the Zika NS1 protein using a variation of SELEX methodology performed on magnetic beads. Seven rounds of selection were applied, reducing the protein target concentration in each round, to a gradient varying from 500 nM on the first cycle to 1 nM on the last, and an aptamer pool was selected. This selected pool had a superior affinity for the target protein than the original library, suggesting successful enrichment, and aptamers were cloned, sequenced, and tested for their binding properties. One of the selected aptamers, denominated aptamer 2, was shown to have the best binding affinity (kd = 24 pM), whereas a different aptamer, aptamer 10, was found to have superior specificity but weaker binding (kd = 134 nM). This pair of aptamers was further optimized and characterized, and their minimum binding domains were determined. Truncated versions of the two aptamers were designed based on M-fold predicted structures, and their binding affinity and specificity were further tested. In terms of specificity, the full-length aptamer 2 showed cross-reactivity when tested against the dengue NS1 protein, whereas aptamer 10 was highly specific, showing no cross-reactivity. The truncated aptamer 2 demonstrated cross-reactivity with all four dengue NS1 serotypes, with a higher affinity for Zika NS1 and decreasing affinities for the dengue NS1 proteins in the order DENV-4 > DENV-1 > DENV-2 > DENV-3. Neither the truncated nor the intact aptamer 10 showed a binding affinity for the DENV NS1 proteins of any of the four serotypes, demonstrating high specificity for the ZIKV NS1 protein, possibly binding in a different domain characteristic of only the NS1 protein of this virus. Neither of the two selected aptamers showed any affinity for nonrelated proteins such as serum proteins (bovine serum albumin or interferon-γ), demonstrating their specificity for the NS1 flavivirus protein. This pair of antibodies was used in an ELASA-type experiment, where aptamer 2 was used as the capture agent due to its superior affinity, whereas aptamer 10, conjugated with biotin, was used for detection, based on the specificity criterion. For tests in buffer, this pair of aptamers performed equally or superiorly to currently used antibody-based immunoassays, with sensitivity ranging from 0.1–1 ng/mL against the 0.2–3 ng/mL described for current immunoassays. When the same experiment was performed in serum, using an aptamer/antibody pair, the detection level increased, but still remained as low as 10 ng/mL, with an absolute specificity for the Zika NS1 protein, showing no detection of the same protein from other flaviviruses such as DENV, WNV, or YFV. Therefore, aptamers were successfully selected against the NS1 protein, and when used as an aptamer/aptamer pair designed based on their properties, or in aptamer/antibody hybrid pairs, can detect the ZIKV NS1 protein in ELASA-type assays with high specificity and sensitivity. The use of aptamer pairs has been limited, and this is one of the exceptional cases where an aptamer pair has been successfully used for the detection of target proteins at levels below the 100 ng/mL mark. Both aptamer/aptamer and aptamer/antibody pairs offer the potential for diagnostic applications in ZIKV infections and possibly other flaviviruses [15].

It is relevant to point out the work of Kim et al. [49], which is based on the development of a diagnostic assay based on aptamer peptides to detect the ZIKV virus in serum and urine. This work is interesting because it is not based on the use of DNA or RNA aptamers selected by SELEX but on the use of what the authors call aptamer peptides. These are small sequences of amino acids (peptides) selected by bioinformatics programs, which the authors call aptamers. The article describes a novel epitope peptide of the ZIKV envelope protein that was predicted using three immune epitope database analysis tools, ABCpred, BCPreds, and Bepipred. A total of 25 peptide aptamers was selected, and their binding ability to the epitopes was evaluated in a molecular docking study. One aptamer peptide was selected and further modified. The modifications consisted of several mutations that generated different peptides, which were also evaluated by docking studies, based on both binding affinity and root-mean-square deviation to increase sensitivity to the ZIKV envelope proteins and decrease sensitivity to the DENV envelope proteins. One of the peptides showed lower energy, satisfying the higher binding affinity requirements. In this work, the authors report that the ZIKV envelope protein (amino acid 1–504) was cloned; the recombinant antigen was produced in *Escherichia coli* and used to make monoclonal antibodies that were subsequently tested experimentally. Peptides and antibodies were conjugated to Eu NPs (europium nanoparticle beads) and experimentally tested using a fluorescence-linked sandwich immunosorbent assay (FLISA). The performance of the peptide-linked sandwich FLISA was evaluated in virus-spiked human serum and urine. In the interaction assays, the authors found that the aptamer peptide was able to detect the Zika protein at a minimum concentration of 0.16 µg/mL and bind to the virus at 1 × 10^5^ tissue culture infective dose (TCID)50/mL. Importantly, the specificity of the test was evaluated using the dengue and Chikungunya viruses, for which there was no binding of the peptides. In FLISA, the performance of the assay significantly increased by at least 5 × 10^4^ TCID50/mL when the virus was diluted in Dulbecco’s modified Eagle’s culture media. When the virus was diluted in the serum, although the signal strength of the test decreased, the detected titer remained 5 × 10^4^ TCID50/mL. Interesting data demonstrated by the authors revealed that these results were obtained when pairs of peptides for capture and development were used in the assay, but a significantly lower titer of viruses is detected when pairs of monoclonal antibodies were used (1 × 10^6^ TCID50/mL titer). When evaluating the titer in urine, the authors found no differences when using the pair of aptamer peptides or the pair of monoclonal antibodies; both the titers detected were 1 × 10^4^ TCID50/mL. Interestingly, the authors commit in their conclusions that an advantage of using these aptamer peptides, instead of DNA aptamers, is the lack of quality clinical samples; the DNA aptamers used in aptamer-based ELISA assays have been reported to detect 1 ng/mL in 10% serum and monoclonal antibodies are still needed in the tests.

In parallel to strategies for the development of new techniques for the human diagnosis of arboviruses, Bosak et al. [50] described an interesting method of mosquito surveillance and control. Their work is based on aptamers conjugated to AuNPs for the development of compact, lightweight, robust, self-contained tools to identify and detect vector mosquitoes and arboviruses via colorimetric readouts. The colorimetric reading of these Apt–AuNP conjugates is based on an aggregation that occurs in the solution of the gold nanoparticles. When the target is not present, Apt–AuNPs are dispersed and the solution is red. When the target is present, the aptamers coupled to AuNPs undergo a conformational change, thus inducing the aggregation of said conjugates, and the color of the solution changes from red to blue/purple. In this work, aptamers selected against an *A. aegypti* salivary gland extract were coupled with AuNPs and tested. The Apt–AuNPs were able to recognize up to 10 ng of protein from the *A. aegypti* salivary gland extract as well as 15 ng of recombinant *A. albopictus* D7 protein. Another approach was to select and test several aptamers against recombinant ZIKV protein E. The aptamers were conjugated to AuNPs, and the aggregation, measured by absorbance, was positive when concentrations of 5 to 50 nM of protein were tested. When Apt–AuNPs were tested against the intact virus, the amount of 10^5^ PFU of active ZIKV was detected in the test. In this work, the tests were performed in a buffer solution, but the authors reported that the ultimate goal was to detect these targets via end-use card-based diagnostics or to observe a color change of the mosquito abdomen after imbibing the Apt–AuNP solution. These preliminary results indicate that the use of this technique, employing Apt–AuNP-based colorimetric measurements, can be a useful tool for diagnosing mosquitoes carrying arboviruses.

Aptamers capable of blocking enzyme MTase action have also been studied as the cap methylation of flavivirus RNA by MTase is an essential process of viral replication. The flavivirus replicates in the cytoplasm, and subsequently, it uses its own enzyme for RNA capping. Flavivirus MTase transfers a methyl group from S-adenosyl–L-methionine to both the N-7 position on the cap and the 2′-OH position of ribose on the first transcribed nucleotide. The flavivirus MTase domain is located at the N-terminal region of the NS5 protein and specifically methylates viral genome RNA by recognition of a specific sequence and structure of its 5′ untranslated region. This enzyme also presents guanylyl transferase activity. Han and Lee [51] commented that the disturbance of viral cap methylation by targeting JEV Mtase might be a potential approach to developing anti-JEV therapy. The targets JEV MTase, replicase, and NS5 were obtained by PCR from a plasmid containing the full-length cDNA of JEV. Human mRNA cap guanine–N-7 methyltransferase (hRNMT) was obtained from HEK293 cells by RT–PCR. The genes were cloned, and the His-tag recombinant proteins were expressed and purified. For SELEX, the authors used a modified RNA library containing 2′-O-methyl pyrimidine nucleotides and, after 15 rounds of selection, the cDNA was cloned. The G2 aptamer bound to MTase and NS5, but not to JEV replicase domain located at the C-terminus of NS5 and hRNMT, indicating that the G2 aptamer has shown specificity for the JEV MTase domain. The G2 aptamer showed a high affinity against MTase (Kd ~ 12.78 ± 1.907 nM) and NS5 (Kd ~11.04 ± 1.763 nM). In another experiment, it was proven that purified recombinant JEV MTase could specifically methylate the N-7 position on the cap of JEV 5′-UTR RNA but not that of HCV 50UTR RNA through an in vitro methylation assay. The methylation of the N-7 position on the cap of JEV 5′-TR RNA by MTase was efficiently inhibited by the G2 aptamer. Since the G2 aptamer competes against the interaction between JEV 5′-UTR RNA and MTase, competitive binding to JEV MTase by the G2 aptamer may inhibit viral substrate methylation, as was subsequently proven. When JEV 5′-UTR RNAs with m7GpppAOH or GpppAOH were used as 2′-OH methylation substrates in the presence of the G2 aptamer, the G2 aptamer efficiently suppressed MTase-mediated 2′-OH methylation of the ribose on the first nucleotide of both capped substrate JEV RNAs, up to approximately 65% and 79% respectively, demonstrating that although the G2 aptamer is not a suitable substrate for JEV MTase, it is capable of inhibiting the action of this enzyme, blocking methylation of the N-7 as well as the 2′-OH groups. It was subsequently proven that the aptamer can inhibit the production of JEV in the host cell. To test this hypothesis, the authors used a cotransfection experiment of BHK-21 cells with the aptamer and viral genome RNA purified from JEV viral particles. The JEV genome RNA production was suppressed by 73% and 60% after 24 and 48 h, respectively. Subsequently, the aptamer was optimized through truncation, and the 24-mer truncated G2 aptamer showed similar affinity and specificity to JEV MTase and continued successfully inhibiting viral replication. JEV MTase is responsible for methylating the viral RNA cap, and its inhibition, without affecting the host cell’s methylation processes, can be an interesting approach in the development of antiviral therapeutic strategies. This particular aptamer was shown to possess such properties, binding and inhibiting JEV MTase, but not hRNMT, thus specifically inhibiting viral methylation, making it a potential antiviral agent for the treatment of JEV. Other flaviviruses, including DENV, WNV, YFV, and ZIKV, also have an MTase domain on the NS5 protein, and perhaps this rationale can be applied to these arboviruses. Thus, Han and Lee [51] and Jung et al. [52] demonstrated the development of RNA aptamers for successful MTase inhibition in DENV. They used MTase produced from each of the DENV serotypes as a selection target, obtained by RT–PCR from viral genomic RNA and cloned into a pQE-80L expression vector. Both viral MTase and human hRNMT were produced as His-tag recombinant proteins and purified by Ni-NTA chromatography. The DENV MTase was exposed to a fluoro-modified RNA library for 13 successive rounds of selection and amplification, which yielded binding aptamers characterized in a gel shift assay. All but one of the selected aptamers bound to DENV2 MTase. Subsequently, the ability of the selected aptamers to inhibit the methylation of the capped DENV2 RNA by the viral MTase was verified in an in-vitro methylation inhibition assay. Four aptamer groups were shown to successfully inhibit N-7 methylation of the viral RNA cap, whereas one of the selected groups of aptamers failed to do so despite its high affinity for the viral MTase target. Two of the aptamers that showed almost complete inhibition of methylation were optimized through truncation, but only aptamer 3 (45-mer) maintained its binding specificity and avidity to the target after truncation, as well as its inhibitory action against all DENV serotypes. This 20-fluoro aptamer was an appropriate therapeutic candidate, combining nuclease resistance, an appropriate size for chemical synthesis, flexibility, and reduced immunogenicity, with high affinity, specificity, and inhibitory activity against its viral MTase target. As mentioned above for JEV MTase inhibitor selection, such an inhibitor would be valuable as a therapeutic agent if it could block viral methylation without affecting that of the host cell. Thus, this DENV MTase specific aptamer was also tested for its specificity for DENV against the MTase of other flaviviruses, as well as against the host hRNMT protein, showing that this molecule was specific for its target protein and thus useful as an antiviral agent against DENV.

## 6. Conclusions

In this review, we have shown that aptamers can be selected against a variety of viral targets of arboviruses of clinical and public health relevance, from viral capsids to envelop to nucleoproteins and their fragments. A number of complicated selection strategies can be optimized for diagnostic or therapeutic applications, whereby aptamers with the lowest dissociation constants or functional aptamers with neutralizing or enzymatic inhibition potential can and have been successfully selected.

Such aptamers have been applied in diagnostic approaches that range from electrochemical or fluorescence biosensors to serological type assays such as ELASA or sandwich ELISA, alone, as a pair of aptamers, or in combination with monoclonal antibodies, with promising results. Similarly, several aptamers have been developed for therapeutic approaches, aiming at directly neutralizing the virus in question, or inhibiting the action of proteins or enzymes relevant for viral replication, thereby offering a therapeutic strategy in moments when no vaccine or other treatment is available. The appeal of a Zika therapeutic or prevention agent for pregnant women, which could avoid the potential for microcephaly or neurological damage to the baby, is an obvious example, as is the treatment for severe cases of arbovirus infections that lead to encephalitis or even death.

Whether for early diagnosis or treatment, aptamers have been raised against a number of arboviruses and have shown promising results, high affinities and specificities, and other beneficial features such as increased stability and reduced size that can be exploited clinically and bring new and highly demanded products to the market.

## Abbreviations

Au NPs	gold nanoparticles
CCHF	Crimean Congo hemorrhagic fever
CHIKV	Chikungunya virus
DENV	Dengue virus
DNA	deoxyribonucleic acid
EDIII	envelope protein domain III
ELASA	enzyme-linked aptamer sorbent assay
ELISA	Enzyme-linked immunosorbent assay
Eu NP	europium nanoparticle beads
FLISA	fluorescence-linked sandwich immunosorbent assay
HBV	Hepatitis B virus
HCV	Hepatitis C virus
HIV-1	Human Immunodeficiency Virus type 1
HPV	Human papillomavirus
hRNMT	human mRNA cap guanine–N7 methyltransferase
ICT	immunochromatographic test
IgG/IgM	Immunoglobulin G and M
IMA	immuno-magnetic agglutination assay
JEV	Japanese encephalitis virus
Kd	equilibrium dissociation constants
kDa	kilodalton
LF	lateral flow
MAYV	Mayaro virus
MPA	3-mercaptopropionic acid
N protein	nucleocapsid protein
NS	nonstructural
OROV	Oropouche virus
PCR	polymerase chain reaction
PFU	Plaque-forming unit
PoC	point of care
PK cell	pig embryo kidney cell
RNA	ribonucleic acid
RVFV	Rift Valley fever virus
SAMNs	surface active maghemite nanoparticles
SARS	Severe Acute Respiratory Syndrome
SELEX	Systematic Evolution of Ligands by Exponential Enrichment
SLEV	Saint Louis encephalitis virus
TBEV	Tick-borne encephalitis virus
TCID	tissue culture infective dose
WNV	West Nile virus
ZIKV	Zika virus
YFV	Yellow Fever virus
